# Clinic of Professor Dunglison

**Published:** 1845-08

**Authors:** Samuel G. White

**Affiliations:** Georgia


### CLINICAL LECTURES AND REPORTS.

### PHILADELPHIA HOSPITAL.

*Saturday, February* 8, 1845.

### CLINIC OF PROFESSOR DUNGLISON.

Reported by Dr. Samuel G. White, of Georgia.

The attention of the clinical class was first directed to the case of CHOREA, the history of which was detailed at the previous lecture. The patient has been under the use of the ferri subcarbonas, in doses of twenty grains three times daily, and a purgative has been given twice a week. Under this joint tonic and cathartic treatment he has considerably improved; locomotion being better executed, and the motions of the limb diminished. It will, therefore, be continued, increasing the dose of the iron to twenty-five or thirty grains. This may be successful in effecting a cure; the prognosis is, however, always less formidable in adults, than in individuals below the age of puberty.

The Professor then proceeded to make some observations on

### CHRONIC CUTANEOUS DISEASES.

Before doing so, however, he introduced a female, labouring under a most loathsome affection of the skin of the head and the body generally. She has been for a considerable length of time in the lunatic wards, but entered the hospital to be treated for syphilis, on which *vice* the cutaneous disease is probably somewhat dependent.

Great difficulty has always existed in properly classifying diseases of the skin. The classifications adopted by different authors are very various. By some they have been supposed to be inflammatory in their character, but this division is objectionable, as it is doubtful if the majority have any connection with that morbid process of nutrition.

The lecturer believes, however, that all chronic cutaneous affections are dependent on diseased nutrition, which must be modified before a cure can be effected. The blood itself may not be diseased, but the tissues which it bathes are, and in all probability both are implicated. He prefers that classification which is based on the elementary form of the eruption, as the vesicular, pustular, bullar, tubercular, exanthematous, &c. This seems little liable to objection to the teacher and student, and is most generally adopted.

The Professor remarked, that these diseases might be studied from plates so as to form an idea of their general characters; but from personal experience he could say, that the application, in practice, of the information thus obtained, is still difficult. Practically, the differential diagnosis is not of great account, as the plan of treatment applicable to one case is appropriate for all.

In the present instance, from the elementary form of the eruption, which is pustular, and from its special characters, the Professor has no hesitation in pronouncing it to be IMPETIGO SPARSA, the eruption being scattered over the whole surface. When collected in patches of different forms, it is IMPETIGO FIGURATA. This cutaneous affection may be confounded with porrigo; but the disease in the latter is more confined to the head, and does not generally extend to the whole body, as in this case.

The seat of the eruption is the corium, but, as before stated, the disease is connected with a depraved condition of the nutritive process. Every variety of chronic cutaneous disease is referable, the lecturer thinks, to this cause, and he accordingly always has recourse, in the treatment, to appropriate eutrophics.

In the present case, iodine was first directed, which was ordered to be pushed so far, as, if possible, to produce iodism, or the decided evidences of its effects on the constitution. By this remedy it was endeavoured to modify the condition of the blood, and, through this, to make an impression on the affected tissues which might cause them to take on a new action. In addition to this, a topical application of the ointment of iodide of sulphur was made. This compound of iodine and sulphur the lecturer has found extremely beneficial in numerous cases. But it is very important, before applying any remedy, that the incrustations, which form on the diseased surfaces, should be removed, otherwise no good will result. This may generally be effected by bathing the parts with warm Castile soap and water, and after the scales have once been removed, care should be taken to prevent their recurrence. If necessary, the parts should be cleansed six or seven times daily: for if this be neglected, no ointment, or other local application, can be of the least advantage. In hospitals it is not difficult to have this attended to, as a person may be appointed for the express purpose of removing the concretions as they form; but, in private practice, it constitutes the great difficulty in the management.

The plan of treatment just indicated as appropriate to impetigo, the Professor regards as applicable to every case of chronic cutaneous disease. The remedies employed to fulfil these indications are numerous. The character of the topical applications will of course be modified by the existence, or not, of inflammation in the parts. When there is none, gently stimulating ointments will often prove highly beneficial; but if there be inflammation, the applications should be of a soothing nature,—as warm fomentations,—and after reducing all excitement, others of a different kind may be resorted to. As some vice exists, however, in the system, in the vast majority of cases, it must be corrected before any permanent benefit can result from external means. The main object, therefore, should be to administer those remedies which will modify nutrition.

The iodide of potassium is generally had recourse to, and it will, in most instances, answer every purpose. Should it fail, however, other preparations may be substituted.

Recently, "Donovan's solution," Liquor Hydrargyri et Arsenici Iodidi, has been highly recommended in the treatment of chronic cutaneous diseases in general. It combines the action of three potent articles, and may be productive of great benefit in some cases, where the preparations of iodine, mercury, and arsenic have failed, when given alone. It may be prescribed in doses of fifteen minims, three times a day, gradually increasing the dose. With the same intention, the alkalies have been given in large doses. They, also, enter the circulation, modify the condition of the blood, and thus prove beneficial. They have, likewise, been used as topical applications, when moderate stimulation of the parts has been deemed necessary. The tar ointment—unguentum picis—was formerly much employed, but it has fallen into comparative disuse, since its active property has been found to reside principally in creasote.

The *unguentum creasoti* may be used with good effects, where it is desirable to produce any excitement, but the ointment of iodide of sulphur is preferable in most cases.

In every instance, the application of topical agents should be conjoined with the administration of internal remedies. But it should be remembered, that whatever mode of treatment be adopted, it must be continued for weeks, or even months. The Professor here observed, that chronic cutaneous diseases are always very difficult of management, and particularly so in private practice. Great credit often accrues to the practitioner, should he succeed in treating an obstinate case successfully; and he referred to the case of a gentleman, now in extensive practice in a neighbouring city, the foundation of which was partly laid by his having been successful in removing an inveterate cutaneous disease, the cause of much disfiguration and annoyance, by one of the preparations of arsenic. A great secret in the treatment is—perseverance in the use of the remedies employed,—and in all cases, as before remarked, care should be taken to remove and prevent the re-formation of incrustations, that will assuredly interfere with the action of topical applications.

In conclusion, the Professor remarked, that as all chronic cutaneous diseases are referable to morbid nutrition, they might with great propriety be classed with the cachexiæ. Convenience, however, results from considering them as a distinct class of affections, and therefore it is well to retain them separate.

The attention of the class was then directed to the consideration of a most interesting case. From the first examination, the lecturer pronounced it to be one of

STRICTURE OF SOME PORTION OF THE INTESTINAL CANAL, and agglutination of the intestines, from chronic peritonitis.

The patient—a man—æt. 44, entered the hospital on the 31st ult. The history of his case, as correctly as could be obtained, is, that about nine weeks since, after eating a large quantity of persimmons, he was seized with violent pain in the stomach, and vomiting. These pains continued for two days, and were so severe as to confine him to bed. They seemed to be paroxysmal, recurring at intervals of half an hour, and were accompanied with costiveness. The pain continued for about two days; and the bowels were not opened until the expiration of that time. He now felt able to get up, and go about, but not to attend to his work. About three weeks since, the pains became more violent, and continued up to the time of his admission into the hospital. They are intermittent, with about half an hour's interval of comparative ease. When first seen he had hiccough: the pulse was not much more frequent than natural; tongue slightly coated. When affected with a paroxysm of pain, the intestines appeared to be thrown into spasm, and to form knots, which were apparent through the parietes of the abdomen. The folds seemed to have become adherent, probably from resulting chronic peritonitis.

When Professor Dunglison first saw him, he presented these phenomena, which were referred by him to some source of obstruction in the intestinal canal: and he directed the stomach tube to be carried into the colon, and soapsuds to be thrown in, so as to distend it. This had not been accomplished effectually, and did not occasion much fecal discharge; an enema of oil of turpentine and mixture of assafœtida was then thrown up, which brought away a considerable amount of indurated feculent matter, with much flatus. He still suffers, however, from the paroxysms of pain, and is exceedingly feeble, the voice being much altered, and the appearance highly anæmic, with great emaciation.

The patient was brought in on his bed and exhibited to the class. The abdomen was protuberant; and much air was contained in the intestines. Whilst the lecturer gently percussed the intestines, to exhibit the sound rendered over various parts, one of the spasms of the intestines supervened; when the folds of intestine were distinctly seen through the parietes of the abdomen; and pressure indicated the existence of a knotty hardness here and there. The affection being seated in the centre of the abdomen and hypogastric region, the small intestines would appear to be the intestines implicated; but the lecturer stated that he had frequently seen the sigmoid flexure much displaced in cases of stricture near its rectal termination. He had seen a figured evacuation from this individual, and had noticed that it had not the usual dimensions, and was much flattened on one side; whence he inferred that stricture existed, probably in the colon. He was in hopes, however, that it might be caused by some temporary narrowing of the canal by hardened fæces, and he would treat the disease altogether by the rectum, throwing up soapsuds so as to fill the colon and cæcum, and soften any obstruction that could be acted upon in this manner. No remedy would be given by the mouth; and the diet would be strictly regulated. The effects produced by this treatment would be reported to the class at the next lecture.

The Professor concluded the lecture with a few observations on

EPILEPSY.

This disease was formerly supposed to be incurable, and superstitious ideas were often associated with it, as indicated by some of its appellations, Morbus sacer, M. divinus, &c. At the present day, such cases, when inveterate, are usually, both in this country and Europe, assigned to a particular ward, where very little or nothing is done for them, and the *epileptic* is regarded as nearly synonymous with the *incurable* ward. Where the disease has continued for a length of time, very little benefit can be obtained from treatment; but this does not justify the almost total neglect, so often observed, of these cases.

The same benefit is not received by time, in epileptic, as in paraplegic or hemiplegic patients; and it is somewhat strange that such should be the case, inasmuch as the latter are always dependent on some structural lesion of the nervous centres.

The pathology of epilepsy is not as yet understood. There is some modification of the neurine, inappreciable by our senses, which occasions the various phenomena that characterize it. Whenever opportunities for examination have occurred, nothing satisfactory has been discovered. Sometimes there may have been a depressed bone, an exostosis, or clot of blood, or other compressing agent; but the ossifications of the membranes and tumours, to which it is sometimes ascribed, the lecturer considered, may often be regarded as epiphenomena, or occurrences in the course of the disease.

Epiplesy has been divided into three stages, but the division is not important, as it is based upon differences in degree, rather than upon any essential difference in the affection. In the first, and mildest stage, there is simple loss of sensation and intelligence, or *absence.* The second variety is characterized by a greater disturbance of the nervous system, as depravation of the senses, confusion of mind, or slight convulsions, but the individual does not fall down. This is the *petit-mal* of the French authors. The third variety, and perhaps the only one which deserves the name of epilepsy, is expressed by marked symptoms; the patient shrieks out commonly, becomes insensible, falls down, and, during the continuance of the paroxysm, is totally devoid of consciousness. The convulsions of the extremities and face are marked and often violent.

The attacks may or may not be preceded by evidences of its approach. They occur most frequently in the night, whilst the individual is asleep. This has been supposed to be owing to an alteration in the circulation of the brain, during the horizontal position, more blood being conveyed to it. The Professor, however, believes it to be dependent on a peculiar modification of the condition of that organ during sleep, which is favourable to the occurrence of the convulsions.

The patient is generally ignorant of having had the attack during the night, except from the feeling of exhaustion it produces in the morning, or from some injury he may have inflicted on himself during the paroxysm.

As epilepsy is connected with an inappreciable condition of the neurine, nothing is known of its remote causes; and hence it is classed amongst the neuroses.

The exciting causes are, generally, the presence of some indigestible or irritating matters in the stomach, the impression of which is conveyed through the nerves to the true spinal axis, and thence to the encephalon. Convulsions, indeed, are of two kinds—*centric*, or those whose cause is seated immediately in the nervous centres; and *eccentric*, or those caused by irritations in remote parts, which are transmitted along the nerves to the sensorium, through the spinal medulla. Of the latter character, are the convulsions affecting children during the process of dentition. In probably the vast majority of cases of epilepsy, the cause is centric, and hence unfavourable.

Formerly, it was supposed that the moon had some influence in the production of epileptic paroxysms; and it was believed, that they were more frequent in some, than in other phases of that luminary. The same idea was prevalent in regard to maniacal patients; and hence the term "lunatic," applied to those cases. But this opinion has been conclusively disproved by repeated observations. It has been ascertained, that if the apartment of the patient be darkened, so as to exclude the light of the moon, maniacal exacerbations are not more frequent during the period of full moon than at any other. If, however, the light be permitted to enter the room, it acts as an irritant, and may thus excite a paroxysm. The same may be said of the influence of solar heat. It is an irritant, and hence maniacal exacerbations are frequent at the summer solstice, when great heat and much light prevail—both of which are powerful excitants.

The prognosis, in the greater number of instances, is unfavourable as to ultimate recovery, if the disease has been of long continuance. It usually impairs the intellect to a great extent, producing dementia. Some of the patients exhibited to the class well exemplified this statement.

The Professor here made a slight digression to explain the difference between dementia, idiocy, and mania, and melancholy. The first being that state in which the intellect is impaired or destroyed, after having been originally of average character, or even above the average. Idiocy is where the impairment or privation was congenital; and in mania and melancholy the mind is more or less perverted on all subjects, the individual being wild and incoherent, or gloomy and drooping; and this, often, on one train of thought, constituting monomania.

Epilepsy is most likely to be confounded with hysteria, but the two affections are usually easily discriminated. In the epileptic paroxysm there is entire unconsciousness; whereas, in the hysterical, the patient is aware of all that is going on around him. Sometimes, however, the affections very closely resemble one another, and demand close attention to distinguish them.

It has been already stated, that this disease is ordinarily considered incurable, so that often nothing is done; but there seems no good reason why we should not endeavour to treat it, as the disturbance of the nervous system, so far as is known, is functional, or at all events the organic lesion is so difficult of appreciation as to prevent it from being understood.

Considerable discrepancy exists as to the proper mode of treating this affection. Some regarding it as congestive, and, at times, inflammatory, have recourse to blood-letting, either general or local; but this practice is seldom warrantable, as there is rarely or never inflammation of the brain or its meninges. The Professor considers it to consist essentially in a preternatural impressibility of the nervous system, which generally requires to be treated with tonics, and such articles as may invigorate the constitution. Amongst the tonics, the nitrate of silver is perhaps most frequently employed, and it is often attended with benefit. The lecturer remarked, that he had derived more good from its administration than from any other article of the materia medica, but he would rely more on properly regulated hygienic regimen, than on any therapeutical agency.

All the mineral tonics, as the preparations of zinc, iron, copper, &c., have also been prescribed. Some years since it was customary to give large quantities of indigo; but this practice is now greatly abandoned. Its beneficial influence was probably dependent on the new impression made on the nervous system, by its presence in the stomach. Medically, it is, perhaps, not better than so much saw dust, or any other indigestible substance. Recently, mugwort, *artemisia vulgaris*, has been greatly lauded in Germany, in the treatment of epilepsy, but it probably has no advantage over the articles already alluded to.

The Prussian blue, and a great variety of tonics, have at some time or other been used. All these remedies, however, have but a temporary effect, and exert no curative influence.

The proper period for the administration of our medicines is in the interval between the paroxysms; but these are so variable, and often so protracted, that it is difficult to persuade the patient to subject himself to any plan of medication, and if he could be persuaded to do so, no accurate estimate could be formed of its influence.

Professor Dunglison considers, that attention should be principally paid, as in the convulsions of children, to avoid or remove exciting causes. Where any mechanical cause exists—as a depressed fracture of one of the cranial bones, or an exostosis seems to be diagnosticated,—benefit maybe, and has been, derived from a surgical operation. But no operation would be justifiable, except we were perfectly certain of the existence of such a lesion. The risk of exciting dangerous encephalitis should never be incurred by searching for any supposed mechanical cause.

During the paroxysm, little more can be done than to protect the patient against himself. There is great danger of the tongue being injured by the teeth: this may be avoided by placing a cork, or other material, between them.

The young practitioner ought to be acquainted with all these expedients, and his practical skill and tact are often estimated in this manner. The Professor well recollected being struck by the simple mode employed by Sir Astley Cooper, in a case of great infiltration of the tongue—the effect of mercurial ptyalism. The organ was protruded from the mouth, and the jaw had closed upon it so that the protruded portion was black and cold. By seizing hold of the tongue enveloped in a cloth, and pressing upon it, the fluid was forced from the tip of the tongue towards the root; and as soon as it could be passed into the mouth, Sir Astley placed a piece of gauze over the mouth, which was fastened behind the neck. In this way the protrusion was effectually prevented, whilst the patient could breathe readily.

The Professor here stated, that in the convulsions of children he rarely found it necessary to abstract blood. He considers them to be generally dependent on extraordinary impressibility of the nervous system, peculiar to the age, and directs his remedies accordingly. They are, for the most part, eccentric, or are excited by the presence in the stomach of some indigestible matter, or by the irritation of dentition. When such offending causes exist, they should be removed by a gentle emetic, and the gums should be freely divided.

Sometimes these convulsions appear hereditary, and bear a striking resemblance to paroxysms of epilepsy, but they generally disappear under the evolutions that take place in the system at puberty, or even before that epoch.

